# Authors’ reply: Toward integrated, fewer-biopsy surveillance after heart transplantation

**DOI:** 10.1093/ehjci/jeag231

**Published:** 2026-09-07

**Authors:** Marko Taipale, Tiina Ojala

**Affiliations:** Department of Pediatric Cardiology, New Children’s Hospital, Pediatric Research Center, University of Helsinki and Helsinki University Hospital, PO Box 281, FI-00029 HUS, Helsinki, Finland; Department of Pediatric Cardiology, New Children’s Hospital, Pediatric Research Center, University of Helsinki and Helsinki University Hospital, PO Box 281, FI-00029 HUS, Helsinki, Finland

We thank Choudhary and colleagues for their thoughtful Discussion Forum and largely share their vision of a risk-adaptive surveillance strategy after heart transplantation. As there is no single true gold standard for allograft rejection, we believe the aim should not be to replace one gatekeeper with another, but to integrate complementary methods that capture different aspects of the rejection process and enable earlier intervention.

This principle was reflected in our study design, in which acute rejection was defined using a composite reference incorporating endomyocardial biopsy (EMB), clinical or echocardiographic findings, and donor-derived cell-free DNA (dd-cfDNA).^1,2^ Models based on isolated criteria were less accurate than those using the composite definition.

A practical surveillance pathway could begin with a comprehensive baseline assessment including cardiac magnetic resonance imaging (CMR), dd-cfDNA, clinical status, and donor-specific antibodies. Longitudinal surveillance could then be primarily CMR-based, dd-cfDNA-based, or combine both, depending on local resources. Where CMR capacity is limited, dd-cfDNA could serve as the initial screening tool, with an abnormal or rising result prompting CMR. In this setting, CMR would primarily replace EMB as the next diagnostic step. Conversely, where imaging capacity allows, CMR could serve as the primary surveillance modality, with dd-cfDNA used complementarily. EMB would be reserved for discordant findings, diagnostic uncertainty, or situations in which histological characterization is needed to guide treatment.

CMR and EMB provide different but complementary information. EMB offers detailed cellular characterization from a small myocardial sample, whereas global CMR mapping can assess the extent and spatial distribution of myocardial injury and allow longitudinal evaluation of treatment response. Moreover, within-patient changes in CMR or biomarker findings may be clinically important even without crossing fixed thresholds. Our study therefore evaluated cut-off values as a necessary first step, while future work should determine how absolute thresholds and individual trajectories can best be combined.

We agree that multicentre validation is essential. The goal should not be a biopsy-free strategy at any cost, but a safer, fewer-biopsy approach in which CMR, dd-cfDNA, clinical assessment, and EMB are used according to their respective strengths. Ultimately, artificial intelligence-driven multimodal assessment may help integrate imaging, biomarker, clinical, and histological data to support earlier detection of rejection and more individualized follow-up.

## Data Availability

No new data were generated or analysed in support of this article.
